# Neutrophil Development, Migration, and Function in Teleost Fish

**DOI:** 10.3390/biology4040715

**Published:** 2015-11-06

**Authors:** Jeffrey J. Havixbeck, Daniel R. Barreda

**Affiliations:** 1 Department of Biological Sciences, University of Alberta, Edmonton, AB T6G2P5, Canada; E-Mail: havixbec@ualberta.ca; 2 Department of Agricultural, Food and Nutritional Science, University of Alberta, Edmonton, AB T6G2P5, Canada

**Keywords:** neutrophils, teleost fish, inflammation, innate immunity, comparative immunology, inflammatory resolution

## Abstract

It is now widely recognized that neutrophils are sophisticated cells that are critical to host defense and the maintenance of homeostasis. In addition, concepts such as neutrophil plasticity are helping to define the range of phenotypic profiles available to cells in this group and the physiological conditions that contribute to their differentiation. Herein, we discuss key features of the life of a teleost neutrophil including their development, migration to an inflammatory site, and contributions to pathogen killing and the control of acute inflammation. The potent anti-microbial mechanisms elicited by these cells in bony fish are a testament to their long-standing evolutionary contributions in host defense. In addition, recent insights into their active roles in the control of inflammation prior to induction of apoptosis highlight their importance to the maintenance of host integrity in these early vertebrates. Overall, our goal is to summarize recent progress in our understanding of this cell type in teleost fish, and to provide evolutionary context for the contributions of this hematopoietic lineage in host defense and an efficient return to homeostasis following injury or infection.

## 1. Introduction

Teleost neutrophils are terminally-differentiated leukocytes that have evolved to protect the animal host and mount early and potent antimicrobial responses against invading pathogens. They are typically the first leukocytes recruited to an inflammatory site [1] and are capable of eliminating pathogens through multiple complementary mechanisms. Upon activation, neutrophils become powerful killers, utilizing toxic intracellular granules [2,3], the production of reactive oxygen species (ROS) [4,5,6], and deploying neutrophil extracellular traps (NETs) [7,8]. These characteristics are shared between teleost neutrophils and their mammalian counterparts [9,10,11,12,13,14]. However, recent data indicate that neutrophil functions extend beyond their historical role of simple pro-inflammatory foot soldiers, expanding our view for the life and death of this long-standing myeloid contributor. Unique features are also arising in different animal models, pointing to a shift in the contributions of this important cell across evolution. In all, neutrophils are critical to the effectiveness of early host antimicrobial responses, the co-ordination of subsequent adaptive mechanisms, the conservation of host integrity, and the maintenance of homeostasis. In this review we highlight recent advances in our understanding of teleost fish neutrophils, and discuss their functional and regulatory roles during the induction and resolution of inflammation. We will largely focus on the role of neutrophils during acute inflammation to complement other recent reviews on the antimicrobial mechanisms of teleost fish leukocytes [15,16,17,18,19,20,21]. Along with other reviews in this special issue, we hope to highlight exciting new advances in our understanding of teleost immune mechanisms.

## 2. The Life of a Neutrophil

### 2.1. Neutrophil Development

Despite extensive evolutionary divergence between teleost fish and mammals, the molecular pathways governing hematopoiesis have been highly conserved. The presence of teleost kidney hematopoietic stem cells (HSCs) and hematopoietic precursor cells (HPCs) responsible for generating all blood lineages was originally demonstrated using transplantation studies in zebrafish and ginbuna crucian carp. Whole kidney marrow from *gata*^eGFP^ zebrafish or β*-actin*^eGFP^ was transplanted into pre-thymic *gata^−/−^* zebrafish [22] or lethally-irradiated zebrafish, respectively, rescuing the phenotype and producing both lymphoid and myeloid cell types. In addition, HSCs from ginbuna crucian carp renal tubules were capable of engraftment into lethally-irradiated recipients, resulting in long-term production of all hematopoietic cells [23]. These studies add further support for the important contributions of the teleost kidney to myelopoiesis.

Successive waves of primitive and definitive hematopoiesis, occurring in anatomically distinct sites, are characteristic of vertebrate embryos [24]. In fish, myelopoiesis and the development of neutrophils have been studied primarily using the zebrafish model. Primitive hematopoiesis takes place intraembryonically in the intermediate cell mass (ICM) [25]. It is here that expression of the granulocyte-specific marker myeloperoxidase (*mpo*) in zebrafish is first detected at 18 h post-fertilization (hpf) [25]. The detection of *mpo^+^* in the ICM suggests this tissue is capable of producing a small population of myeloid progenitors. From here, HSCs from the claudal hematopoietic tissues seed the major hematopoietic organ of teleost fish, the pronephros, followed by the mesonephros, which is functionally analogous to the mammalian bone marrow. It is in this tissue that the generation of neutrophils occurs from 2 days post-fertilization (dpf) [26,27]. However, it is currently unknown how long embryonic granulocytes from the ICM persist or if they contribute to the granulocyte population that is found in the pronephros at 7 dpf [28]. Over the next several weeks of larval development the hematopoeitic tissue expands; however, development remains restricted to the myeloerythroid cell lineages [28]. In adult zebrafish, hematopoietic tissue is present throughout the kidney and all leukocyte lineages appear to be derived from this tissue.

Studies in mice have confirmed that commitment of pluripotent HSCs to myeloid precursors to mature neutrophils is controlled by both the extracellular cues (growth factors, cytokines, *etc*.) [29,30,31] as well as intracellular transcription factors [31,32,33]. In particular, one of the most important cytokines involved in granulopoiesis, the granulocyte-colony stimulating factor (GCSF), which is known to stimulate the survival, proliferation, differentiation, and function of neutrophil precursors and mature neutrophils. Interestingly, mice lacking GCSFR, or patients constitutively expressing hypomorphic GCSFR mutants, are neutropenic, but still possess mature granulocytes, indicating that both GCSFR-dependent and GCSFR-independent granulopoiesis occur in mammals [34,35]. More broadly, the GCSF/GCSFR pathway functions to maintain neutrophil viability and function within an inflammatory site [36]. A recent study confirmed both GCSFR-dependent and GCSFR-independent pathways are also present in zebrafish [37]. Morpholino-mediated knockdown of *gcsfr* and overexpression of *gcsf* in zebrafish discovered the presence of an anterior population of myeloid cells during hematopoiesis dependent on the GCSF/GCSFR pathway for development and migration. However, a population of myeloid cells found within the posterior domain developed largely independent of this pathway [37]. Unlike GCSF, however, GMCSF and IL-3-like molecules have yet to be identified in fish, making further characterization of granulopoiesis difficult. Alternatively, it may point to differences in the soluble drivers for granulopoiesis in these lower vertebrates.

Hematopoietic stem cell commitment to the myeloid lineage is also heavily dependent of the intricate regulation of transcription factors (TFs). TFs are capable of acting independently, co-operatively, or antagonistically when determining lineage fate decisions. The stem cell leukemia (*scl*) gene has been extensively characterized in both mammals and zebrafish, where it plays a critical role in HSC formation [38,39].

However, much like the examination of teleost cytokines and growth factors involved in hematopoiesis, few studies have thoroughly examined the role of neutrophil TFs involved in granulopoiesis.

### 2.2. Neutrophil Reservoirs

A major difference between teleost and mammals relates to the relative numbers of neutrophils in circulation. Whereas in mammals (e.g., humans and swine) neutrophils represent the most prominent leukocyte in circulation (40%–70% and 30%–40%, respectively), in teleosts, other than rainbow trout, they constitute less than 5% of circulating leukocytes in bony fish [1,40,41,42] In contrast, the kidney has been shown to house prominent neutrophilic populations [1,5,43,44]. Recent indications from our lab further show that the large reservoir of neutrophils in the goldfish hematopoietic kidney can be readily deployed to sites of inflammation [1]. These neutrophils rapidly exit the hematopoietic compartment during the first 12 h post-zymosan administration in the peritoneal cavity. This points to the relative importance of the hematopoietic storage pool of neutrophils for bony fish, likely representing the majority of the mature neutrophils that can traffic to a site of microbial challenge.

### 2.3. Neutrophil Migration

Mature neutrophils must migrate across the sinusoidal endothelium that separates the hematopoietic tissue from the circulation. From there, they are rapidly recruited from the blood to sites of inflammation by chemotactic signals derived from the infectious agents (pathogen-associated molecular patterns) and the host (damage/danger-associated molecular patterns). Delineating exactly how individual neutrophils traffic from the bloodstream to tissues *in vivo* has been difficult as a result of the limitations of available mammalian models. Although there have been significant advances using transgenic mice harbouring fluorescent neutrophils, these models are technically complex and require intricate surgical procedures, as well as intravital microscopy [45]. Recently, the zebrafish has emerged as a powerful vertebrate model to study *in vivo* chemoattractant gradients [46] and neutrophil recruitment [47,48] during inflammation. This organism is amenable to *in vivo* manipulation with excellent optical clarity and good genetic approaches. In addition, the zebrafish shows remarkable conservation of its immune system at cellular and molecular levels [49].

One particular molecule, the small chemokine known as CXCL8 (also known as IL-8), plays a critical role in the recruitment of human neutrophils to the site of inflammation. Among several other functions, CXCL8 is responsible for guiding neutrophils through the tissue matrix until they reach the site of injury. A diverse array of sequences similar to mammalian CXCL8 have been reported in teleost fish and we now know that the majority of these teleost sequences exhibit a functional homology equivalent to mammalian CXCL8. It has been shown that all teleost fish possess at least one form of CXCL8 (CXCL8-l1), while two distinct forms can be found in carp and zebrafish (CXCL8-l1 and CXCL8-12). Both forms appear to be functionally equivalent to mammalian CXCL8 [50]. In addition, a recent study in trout found a third isoform (termed CXCL8_L3), however further characterization is still needed in order to fully determine its function [51]. Several studies using different teleost models have gone on to find increased levels of *cxcl8* mRNA expression at the site of inflammation [1,52,53,54]. Zebrafish *in vivo* models add support for the contributions of CXCL8 to neutrophil migration [52,53,54]. Finally, recombinant CXCL8 molecules from both forms in carp have also been shown to induce chemotaxis in neutrophils at 200 ng/mL *in vitro* [50].

Teleost models have also provided us with the ability to examine the movement of neutrophils using whole animal studies. For example, using an *in vivo* zymosan peritonitis model in goldfish, our lab recently quantified the migration of neutrophils from the hematopoietic compartment to the site of inflammation [1]. The use of *in vivo* imaging in zebrafish has also been an invaluable tool in examining the migration of neutrophils during acute inflammation. *In vivo* studies using *cxcl8* zebrafish morphants displayed reduced neutrophil recruitment during acute inflammation [52]. In addition, Renshaw *et al.* were able to examine fluorescent neutrophils migrating towards the site of injury following tail wounding [47]. Other studies have also taken advantage of this system to examine the movement of neutrophils during inflammation. For example, the movement of PI3K has been examined within individual neutrophils *in vivo* and showed that PI3K is critical for cell motility and must be present at the leading edge during migration [55].

Conventional thinking has assumed neutrophils are one directional, ending with death at the site of inflammation. This dogma has dominated the field of inflammation for many decades. However, a number of more recent studies have challenged this paradigm, suggesting that neutrophils, in some circumstances, can emigrate from damaged tissues by a process of reverse transmigration [48]. *In vivo* time-lapse imaging has shown that neutrophils are capable of retrograde chemotaxis back toward the vasculature, implicating this mechanism as a novel method of inflammatory resolution in zebrafish [48]. This mechanism appears to be conserved in the higher vertebrates, where a specific subset of human neutrophils (CD54^high^/CXCR1^low^) represent long-lived neutrophils that have migrated though an endothelial monolayer and then re-emerged via reverse transmigration [56].

## 3. Neutrophils and the Promotion of Inflammation

Neutrophils are a critical component in the first line of defense against invading pathogens. Multi-receptor recognition of PAMPs and DAMPs define intruding pathogens, resulting in the activation of cellular antimicrobial responses designed to kill infiltrating pathogens. Antimicrobial responses are tailored to the type and location of the pathogen, and can be divided into two main categories: intracellular and extracellular. Intracellular defense mechanisms are designed to provide protection against pathogens found within membrane-enclosed structures. These defenses are not limited to killing pathogens that have been internalized through phagocytosis, but also to provide protection against pathogens actively hiding from humoral immune defenses.

Neutrophils are armed with an extensive antimicrobial arsenal designed to limit the dissemination of a broad range of pathogens. Interestingly, many of the antimicrobial mechanisms present in teleost neutrophils are utilized both as intracellular and extracellular defenses. A key example is the robust production of reactive oxygen (ROS) and nitrogen (NOS) species found in fish neutrophils [43,57,58,59]. Unlike the predominantly intracellular release of ROS and NOS in monocytes and macrophages, teleost neutrophils elicit robust responses both intracellularly and extracellularly [57,60]. Further, antimicrobial and cytotoxic substances stored in neutrophilic granules can be released into the extracellular space (described below), or within the phagosome, where they exert potent antimicrobial actions [61,62].

Defenses towards pathogens within the extracellular space provide a method by which the innate immune system can effectively clear pathogens that have escaped internalization or are too large to be internalized. These responses are generally activated by the presence of microbial products or inflammatory mediators and result in the release of antimicrobial factors into the extracellular space. These soluble products are efficient antimicrobial agents, but their mode of action is generally non-specific [63]. As a result, extracellular release of ROS and NOS also leads to collateral damage of host tissue [57,59]. Unlike the release of reactive oxygen and nitrogen species, products in neutrophilic granules are specifically targeted towards microorganisms and cause little damage to host tissues. Intracellular defenses effectively kill internalized pathogens following phagolysosome fusion, where a toxic degradative environment is established within neutrophils. Targeted production and release of these antimicrobial molecules into membrane-enclosed structures ensures reduced damage to host phagocytes and tissues, while maximizing the degradative potential to internalized pathogens.

### 3.1. Degranulation

As one of the first infiltrating cells into an inflammatory site, neutrophils are armed with a wide arsenal of intracellular and extracellular antimicrobial tools critical to the defense against pathogens. One of the primary extracellular defense mechanisms of neutrophils is the targeted degranulation of cytoplasmic granules containing preformed antimicrobial mediators. Granules isolated from mammalian neutrophils have been shown to contain a vast array of antimicrobial enzymes that include, myeloperoxidase, proteinase-3, cathepsin G and elastase, metalloproteinase, and acidic hydrolases, as well as antimicrobial peptides such as lactoferrin and cathlicidin [63]. While granular contents of teleost neutrophils have not been as thoroughly described, an assay has been developed to quantitate myeloperoxidase degranulation in fish neutrophils [64]. Using this assay, teleost neutrophils have been shown to degranulate following stimulation with various mitogens, zymosan, and *Aeromonas salmonicida* [5,57,64]. Further, degranulation was not affected by the addition of cytochalasin B, indicating this potent mechanism does not require prior phagocytic events. Functional studies and protein analysis have shown that teleost fish express homologues of proteinase-3, cathepsin G, elastase, and azurocidin, helping to further dissect the classical granule contents of teleost neutrophils [65]. However, further examination must be done in order to confirm the expression of these enzymes at a protein level and their functionality, as well as to determine their relative contributions to the effector mechanisms mounted against invading pathogens.

### 3.2. Neutrophil Extracellular Traps (NETs)

Along with degranulation, mammalian neutrophils can also kill extracellular pathogens by NETs [13]. The ultrastructure of NETs consists of smooth filaments with a diameter of ~17 nm [13], containing stacked and likely modified nucleosomes [66]. This backbone is dotted with granular proteins forming globular domains with a diameter of ~50 nm [13]. NETs are capable of multiple mechanisms of action-binding microorganisms, degrading virulence factors, preventing dissemination, and killing bacteria by maintaining a high local concentration of antimicrobial granule components [13]. The production of NETs by teleost neutrophils has been recently described in carp, zebrafish, and fathead minnows [7,8,64,67]. Similarly to mammalian NETs, fish NETs are composed of neutrophil granule proteins associated with extracellular DNA fibres, but not cytoskeleton [7,13,67]. However, much remains to be learned about the contribution of different neutrophilic granule classes to the composition of teleost NETs, as well as the ability of these NETs to prevent pathogen spread and kill invading microorganisms.

### 3.3. Respiratory Burst

The first indication of a respiratory burst was described in mammalian leukocytes in 1933 by Baldridge and Gerard, when it was noted that phagocytosis was associated with increased oxygen consumption [68]. Subsequently, it was found that this increase in oxygen consumption was the result of the formation of superoxide anions [69], in a process catalyzed by NADPH-oxidase [70]. Phylogenetic analysis of the NADPH-oxidase has been described in several teleost fish species, including rainbow trout [71], Japanese pufferfish [72], carp [58], Atlantic salmon [73], zebrafish, and pufferfish [74,75], indicating homology and a common mammalian/teleost ancestor [58]. Further, each lineage has evolved separately, leading to a cluster containing all fish NADPH components [73]. Although fish and mammals share relatively low sequence homology, the functional domains remain highly homologous [58,71,72,73]. Importantly, fish NADPH-oxidase components have been shown to have similar modes of activation and functional activities [58,72,73].

Several studies examining the induction of ROS production in mammalian neutrophils indicate an important role for inflammatory cytokines, including TNF-α [76], IFN-α [77], GCSF, and GMCSF [76]. In addition, neutrophil derived products (PAF and Leukotriene B_4_ (LTB_4_)) have been shown to induce NADPH activity [78]. Although the impact of the majority of these inflammatory molecules have not been examined directly on teleost neutrophils, they have been shown to induce respiratory burst responses in fish phagocytes [79,80,81,82]. Respiratory burst responses in fish neutrophils can also be strongly activated by PAMPs, including LPS, CpG DNA, flagellin, and MDP, which have been shown to induce respiratory burst activity in gilthead seabream [83,84]. Finally, several important fish pathogens, *Aeromonas salmonicida* and *Vibrio anguillarum,* have also been shown to induce potent respiratory burst responses in neutrophils [5,85,86].

Following stimulation with any of these factors, NADPH-oxidase activates through three sequential steps: (i) activation of PKC, (ii) phosphorylation of p47^phox^, and (iii) the production of reactive oxygen intermediates by NADPH-oxidase [73]. While activation pathways are conserved across teleost fish species, the kinetics and strength of the response showed differences across those species studied. For example, a study comparing the production of ROS from carp and ayu neutrophils found that ayu neutrophils spontaneously activated respiratory burst, however the response was not further enhanced by priming [59,87]. In contrast, carp neutrophils displayed low levels of respiratory burst in resting cells that was enhanced in the presence of inflammatory stimuli [59,87]. These observations may highlight a differential level of responsiveness across fish that is partially-driven through the specific requirements and challenges that face each species within its chosen ecological niche. In light of the available results, it will be particularly interesting to go beyond the intrinsic mechanisms that regulate these responses at the cellular level, and assess the differential crosstalk that exists between respiratory burst mechanisms and other antimicrobial host responses across distinct cellular niches. gFurther, we look forward to an increased understanding of the environmental factors that may contribute to the heterogeneity observed in these responses across teleost fish species.

### 3.4. Nitric Oxide

Nitric oxide (NO) is essential to neuronal communications, inhibition of cell proliferation, vasodilation, and intracellular signalling [88,89,90,91]. In addition, it has potent toxic effects and serves as a vital component of antimicrobial defenses. Nitric oxide is formed by the oxidation of l-arginine to l-citrulline by NO synthase (NOS) [92]. Three forms of NOS have been identified in mammals—Endothelial (eNOS), neuronal (nNOS), and inducible (iNOS) [93]. Of these, only the latter has been shown to be involved in immune defense. iNOS was first identified in the goldfish [94], with further characterization in rainbow trout [95] and carp [96]. The identified carp protein shares 57% similarity with human iNOS and contains putative binding sites for heme, tetrahydobiopterin calmodulin, flavine mononucleotide, flavine adenine dinucleotide, and NADPH, all of which represent important sites in mammalian iNOS [96]. Further, iNOS activity has been demonstrated in teleost granulocytes, including neutrophils [96,97]. Similar to mammalian iNOS, transcription appears to require NF-κB [96] following stimulation with cytokines or PAMPs. In addition, the parasitic haemoflagellate, *Trypanoplasma borreli* has also been shown to induce NO production in head kidney neutrophils of carp [43,57]. Nitric oxide has been shown to have potent antimicrobial effects against a number of relevant fish pathogens [98,99,100]. However, little work has been done to characterize nitric oxide responses in teleost neutrophils, and their ability to use NO as a potent antimicrobial mediator.

Nitric oxide release, like the superoxide anions in respiratory burst responses, are not specifically targeted to microorganisms, thus leading to a potentially toxic environment for host tissues. Due to this, NO production must remain tightly regulated. The antioxidant glutathione has also been shown to play a protective role against nitrosative stress [101]. This was most prominent in carp phagocytes that naturally contain higher levels of glutathione compared to other peripheral blood leukocytes [101]. In addition, carp neutrophils have been shown to up-regulate genes involved in the glutathione redox cycle following LPS stimulation, resulting in further protection against the deleterious effects of NO products [101].

## 4. Neutrophils and the Resolution of Inflammation

Resolution of inflammation and the return of tissues to homeostasis are essential to host health and survival. Excessive inflammation is widely recognized as a major component in many chronic diseases, including vascular diseases, metabolic syndrome, and neurological diseases and is, thus, a public health concern. Inflammation functions to neutralize and eliminate pathogenic invaders as well as to clear damaged tissue. Understanding endogenous control points within the inflammatory response could provide us with new perspectives on disease pathogenesis and treatment approaches.

Neutrophils have typically been viewed as necessary, but detrimental, short-lived cells that are recruited early in the inflammatory response. Once the pathogenic threat was eliminated, neutrophils were simply thought to undergo apoptosis, thus signalling resolution mechanisms to begin. However, recent work has altered our preconceived notions of neutrophilic contributions to inflammatory processes. In particular, significant evidence implicates a central role for neutrophils in triggering inflammatory resolution. Such mechanisms involve both metabolic and biochemical crosstalk pathways during the intimate interactions of neutrophils with other cell types at the site of inflammation.

### 4.1. Production of Lipid Mediators

The resolution of inflammation requires a reduction or removal of leukocytes and debris from inflamed sites and the initiation of wound healing mechanisms, enabling a return to homeostasis. It is now understood that uncontrolled inflammation is a unifying component in many diseases and recent evidence indicates that inflammatory resolution is a biosynthetically active process [102] and not simply passive. These new findings direct decision processes wherein acute inflammation, chronic inflammation, or inflammatory resolution outcomes are governed based on the endogenous mechanisms employed to control the magnitude and duration of the response [103,104]. Following an immune challenge, the resolution process is rapidly initiated by cellular pathways that actively biosynthesize local specialized dual-acting anti-inflammatory and pro-resolution lipid mediators, such as lipoxins, resolvins, and protectins [102].

In mammals, an active switch in the lipid mediators released into the exudate accompanies the resolution of inflammation. Initially pro-induction mediators are generated, such as leukotrienes (LT) and prostaglandins (PG), which activate and amplify the cardinal signs of inflammation. Next, two major prostaglandins, PGE_2_ and PGD_2_ gradually induce key enzymes involved in the production of mediators with both anti-inflammatory and pro-resolving function, such as the lipoxins [105], resolvins, and protectins [106,107]. These families of endogenous pro-resolution molecules activate specific mechanisms to promote homeostasis by stimulating and accelerating resolution at the tissue level. Specific lipoxins and members of the resolvin and protectin families are potent stimuli that selectively stop neutrophil infiltration; stimulate nonphlogistic recruitment of monocytes, induce macrophage efferocytosis, increase lymphatic removal of phagocytes, and stimulate expression of antimicrobial defense mechanisms [108,109,110].

Mammalian research has now shown that neutrophils alter their phenotype to produce different lipid mediator profiles depending on the environmental milieu [105,107]. For example, neutrophils found in exudates during inflammatory resolution switch from the production of leukotrienes to lipoxins and resolvins, whereas those in peripheral blood generate and release leukotriene B_4_ upon activation [105]. Interestingly, we have also shown this to be the case in teleost neutrophils. Neutrophils, isolated during the induction of inflammation were found to produce elevated levels of the pro-inflammatory and chemotactic lipid, LTB_4_, whereas neutrophils isolated during the initiation of resolution displayed increased levels of the pro-resolving lipid LXA_4_ [1]. We showed that LTB_4_ increased ROS production in both naïve and pro-inflammatory neutrophils, while LXA_4_ increased the uptake of apoptotic neutrophils by macrophages [1]. In another study, Gomez-Abellan and colleagues further showed that PGD(2) and its metabolites contribute to anti-inflammatory responses in the gilthead seabream [106].

### 4.2. Death of a Neutrophil

Neutrophils are central to the clearance of pathogens. However, despite indications for neutrophil reverse transmigration, the timely entry of neutrophils into apoptosis remains critical for the resolution of inflammation. Apoptosis renders neutrophils unresponsive to extracellular stimuli and leads to the expression of molecules signalling their removal by scavenger macrophages [111,112]. The life and death of neutrophils can be profoundly influenced by signals from the inflammatory milieu [113,114]. Pro-inflammatory mediators, including granulocyte macrophage colony stimulating factor (GM-CSF), IL-8, or bacterial components (LPS, bacterial DNA-CpG motifs, *etc*.) could markedly prolong the longevity of neutrophils, whereas pro-apoptotic stimuli, such as tumour necrosis factor alpha (TNF-α), TNF-related apoptosis-inducing ligand (TRAIL), or Fas ligand can shorten their life span [115,116,117]. Precise control of the neutrophil death program provides an essential balance between their defense functions, appropriate apoptotic clearance, and tissue homeostasis.

A complex network of intracellular death/survival signalling pathways regulates entry into apoptosis ultimately determining the fate of neutrophils. Extensive reviews have already been written regarding the mechanisms of neutrophil apoptosis [114,118,119]. Briefly, intrinsic mechanisms are mediated through mitochondrial pathways and likely involve ROS production, although the mechanism of ROS generation is not well understood in aging neutrophils. The extrinsic pathway triggers apoptosis following ligation of cell surface death receptors (TNF-α, TRAIL receptors or Fas) to form the death-inducing signalling complex. Further, Fas signalling overrides the anti-apoptotic effects of GM-CSF [120].

Neutrophil apoptosis is another critical point in the resolution of inflammation, where many cell types, including “professional” phagocytes such as macrophages, can accomplish the clearance of apoptotic corpses. The process of clearing apoptotic cells can be broken down into a succession of steps, from the recognition of “eat-me signals” on dying cells, internalization and processing of the apoptotic cell, to the downstream consequences following engulfment. Apoptotic cell uptake stimulates the production of lipoxin A_4_, further enhancing the uptake of apoptotic cells [121] and, in conjunction with resolvins and protectins, dominates the resolution phase of the inflammatory response [103]. In addition to autocrine and paracrine effects mediated through cytokines and lipid mediators, *in vitro* and *in vivo* studies have shown that the clearance of apoptotic cells within an inflammatory site decreases the production of pro-inflammatory cytokines, including tumor necrosis factor alpha (TNF-α), IL-6, IL-8, IL-17, IL-23, as well as the lipid mediators PGE_2_ and LTC_4_, promoting the production of anti-inflammatory immune mediators, notably IL-10 and transforming growth factor beta (TGF-β) [122,123,124,125]. Further, the shift in the balance from TNF-α to TGF-β contributes to the quenching of reactive oxygen and nitrogen species [126]. Although, the downstream events following the engulfment of apoptotic neutrophils by macrophages in teleost fish have not been examined to the same extent, several important studies have assessed the functional outcomes of this process. For example, we have found that neutrophils incubated with apoptotic cells are able to down-regulate the generation of ROS in macrophages [1]. In addition, macrophages that have internalized apoptotic neutrophils are capable of down-regulating ROS production in all phagocytes [4]. Interestingly, similarly to mammalian studies, we found lipoxin A_4_ induced an increase in apoptotic cell uptake by macrophages [1].

## 5. Conclusions

**Figure 1:**
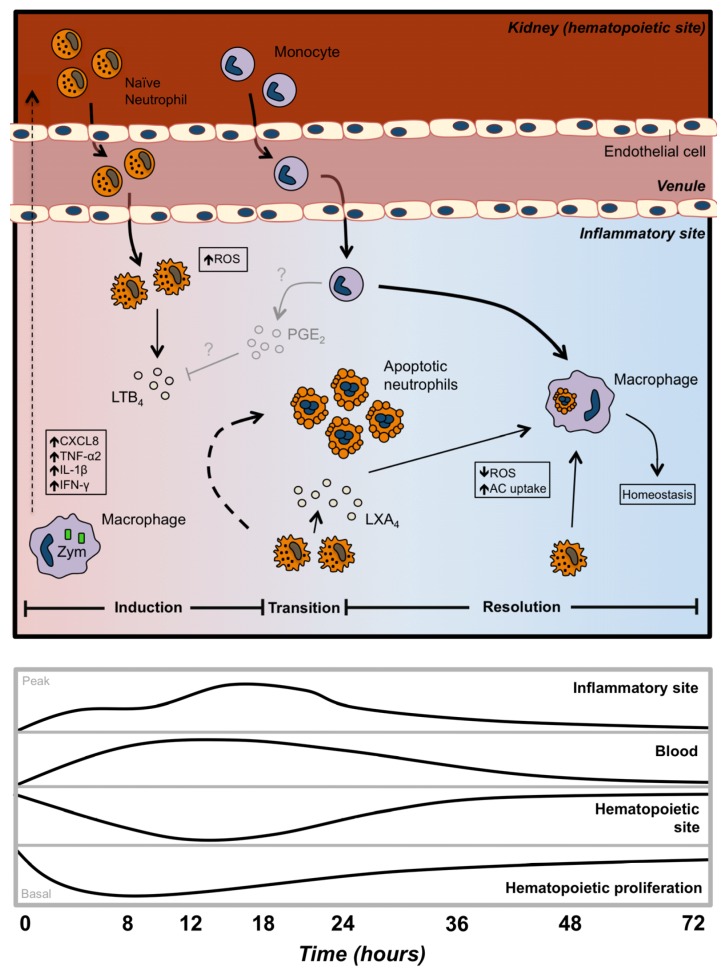
Contributions of neutrophils to the induction, regulation, and resolution of the acute inflammatory response in teleost fish. Peritoneal injection of zymosan results in the release of proinflammatory cytokines, such as CXCL8, TNF-α, IL-1β, and IFN-γ, and chemoattractants like CXCL8 and LTB_4_, inducing the infiltration of neutrophils and other leukocytes. Inflammatory neutrophils enter the site of inflammation and are subsequently activated by the inflammatory milieu, resulting in increased ROS production and the release of more LTB_4_ (pro-inflammatory lipid mediator). As inflammation progresses, a transition from pro-inflammatory to pro-resolution begins to occur (18–24 hpi). Neutrophils then alter their production of lipid mediators to increase the release of LXA_4_, followed by an entry into apoptotic cascades, peaking at 24 hpi. We hypothesize that this switch from LTB4 to LXA4 is mediated, at least in part, by the release of PGE_2_ from local cells (grayed out in model with question marks). The local inflammatory milieu slowly becomes pro-resolving as macrophages are stimulated by LXA_4_, decreasing their production of ROS and increasing their uptake of apoptotic neutrophils. This progression eventually leads to the return to homeostasis by 72 hpi. The relationship between neutrophil production within the kidney hematopoietic tissue, their migration through the blood, and infiltration into the challenge site highlight unique features of the teleost system.

The past decade has seen an impressive expansion in our understanding of the biology of teleost phagocytes, including neutrophils. Despite clear similarities between fish and mammalian acute inflammatory responses, distinct features such as a much reduced number of circulating blood neutrophils presents unique challenges to teleosts for the effective clearance of infectious agents and the resolution of acute inflammation (Figure 1). The capacity of teleost neutrophils to alter their phenotype during the acute inflammation is certainly key to their contributions as effectors and regulators of this response. An increased focus on this phenotypic transition is likely to yield exciting new insights into the evolution of immunity and control mechanisms for inflammation.
